# Supplementary material for: Pyruvate Dehydrogenase Kinase 1 inhibition mediated oxidative phosphorylation enhancement in cartilage promotes osteoarthritis progression
Source: BMC Musculoskelet Disord. 2023 Jul 20;24:597. doi: 10.1186/s12891-023-06585-6 (PMC10357736; doi:10.1186/s12891-023-06585-6)
Supplement: Supplementary file 1 — Additional file 1. [file 12891_2023_6585_MOESM1_ESM.zip › MF.Gsea.1653627316242/GOMF_UBIQUITIN_PROTEIN_TRANSFERASE_REGULATOR_ACTIVITY.html]

Details for gene set GOMF\_UBIQUITIN\_PROTEIN\_TRANSFERASE\_REGULATOR\_ACTIVITY[GSEA]

|  || Dataset | 55457\_collapsed\_to\_symbols.cls.cls #OA\_versus\_normal.cls.cls #OA\_versus\_normal\_repos |
| Phenotype | cls.cls#OA\_versus\_normal\_repos |
| Upregulated in class | normal |
| GeneSet | GOMF\_UBIQUITIN\_PROTEIN\_TRANSFERASE\_REGULATOR\_ACTIVITY |
| Enrichment Score (ES) | -0.58908975 |
| Normalized Enrichment Score (NES) | -1.8034313 |
| Nominal p-value | 0.0035650623 |
| FDR q-value | 0.080317296 |
| FWER p-Value | 0.53 |
Table: GSEA Results Summary

  

Fig 1: Enrichment plot: GOMF\_UBIQUITIN\_PROTEIN\_TRANSFERASE\_REGULATOR\_ACTIVITY      
 Profile of the Running ES Score & Positions of GeneSet Members on the Rank Ordered List

  

| SYMBOL | TITLE | RANK IN GENE LIST | RANK METRIC SCORE | RUNNING ES | CORE ENRICHMENT || 1 | CDC20 | cell division cycle 20 [Source:HGNC Symbol;Acc:HGNC:1723] | 552 | 0.641 | 0.0330 | No |
| 2 | TRIB2 | tribbles pseudokinase 2 [Source:HGNC Symbol;Acc:HGNC:30809] | 2332 | 0.323 | -0.0669 | No |
| 3 | UBE2L3 | ubiquitin conjugating enzyme E2 L3 [Source:HGNC Symbol;Acc:HGNC:12488] | 5428 | 0.038 | -0.3026 | No |
| 4 | FBXO5 | F-box protein 5 [Source:HGNC Symbol;Acc:HGNC:13584] | 8007 | -0.156 | -0.4843 | No |
| 5 | RING1 | ring finger protein 1 [Source:HGNC Symbol;Acc:HGNC:10018] | 8129 | -0.165 | -0.4742 | No |
| 6 | RPL37 | ribosomal protein L37 [Source:HGNC Symbol;Acc:HGNC:10347] | 9265 | -0.253 | -0.5324 | No |
| 7 | FZR1 | fizzy and cell division cycle 20 related 1 [Source:HGNC Symbol;Acc:HGNC:24824] | 9996 | -0.321 | -0.5511 | Yes |
| 8 | RPL5 | ribosomal protein L5 [Source:HGNC Symbol;Acc:HGNC:10360] | 10201 | -0.341 | -0.5266 | Yes |
| 9 | AUP1 | AUP1 lipid droplet regulating VLDL assembly factor [Source:HGNC Symbol;Acc:HGNC:891] | 10487 | -0.372 | -0.5048 | Yes |
| 10 | RPS7 | ribosomal protein S7 [Source:HGNC Symbol;Acc:HGNC:10440] | 10608 | -0.384 | -0.4686 | Yes |
| 11 | BTRC | beta-transducin repeat containing E3 ubiquitin protein ligase [Source:HGNC Symbol;Acc:HGNC:1144] | 10836 | -0.409 | -0.4378 | Yes |
| 12 | TRIB3 | tribbles pseudokinase 3 [Source:HGNC Symbol;Acc:HGNC:16228] | 10839 | -0.409 | -0.3895 | Yes |
| 13 | RPL11 | ribosomal protein L11 [Source:HGNC Symbol;Acc:HGNC:10301] | 11252 | -0.464 | -0.3666 | Yes |
| 14 | GLMN | "glomulin, FKBP associated protein [Source:HGNC Symbol;Acc:HGNC:14373]" | 11476 | -0.498 | -0.3249 | Yes |
| 15 | RPS20 | ribosomal protein S20 [Source:HGNC Symbol;Acc:HGNC:10405] | 11507 | -0.502 | -0.2678 | Yes |
| 16 | FBXW7 | F-box and WD repeat domain containing 7 [Source:HGNC Symbol;Acc:HGNC:16712] | 11524 | -0.505 | -0.2094 | Yes |
| 17 | RPS15 | ribosomal protein S15 [Source:HGNC Symbol;Acc:HGNC:10388] | 11671 | -0.527 | -0.1583 | Yes |
| 18 | TRIB1 | tribbles pseudokinase 1 [Source:HGNC Symbol;Acc:HGNC:16891] | 11924 | -0.577 | -0.1095 | Yes |
| 19 | RPL23 | ribosomal protein L23 [Source:HGNC Symbol;Acc:HGNC:10316] | 12558 | -0.777 | -0.0667 | Yes |
| 20 | CBX8 | chromobox 8 [Source:HGNC Symbol;Acc:HGNC:15962] | 12586 | -0.787 | 0.0244 | Yes |
Table: GSEA details [plain text format]

  

Fig 2: GOMF\_UBIQUITIN\_PROTEIN\_TRANSFERASE\_REGULATOR\_ACTIVITY      
 Blue-Pink O' Gram in the Space of the Analyzed GeneSet

  

Fig 3: GOMF\_UBIQUITIN\_PROTEIN\_TRANSFERASE\_REGULATOR\_ACTIVITY: Random ES distribution      
 Gene set null distribution of ES for **GOMF\_UBIQUITIN\_PROTEIN\_TRANSFERASE\_REGULATOR\_ACTIVITY**
